# Circular RNAs in the Regulation of Oxidative Stress

**DOI:** 10.3389/fphar.2021.697903

**Published:** 2021-07-27

**Authors:** Yao Zhang, Yu Chen, Yue Wan, Yueshui Zhao, Qinglian Wen, Xiaolong Tang, Jing Shen, Xu Wu, Mingxing Li, Xiang Li, Jing Li, Wanping Li, Zhangang Xiao, Fukuan Du

**Affiliations:** ^1^ Laboratory of Molecular Pharmacology, Department of Pharmacology, School of Pharmacy, Southwest Medical University, Luzhou, China; ^2^ South Sichuan Institute of Translational Medicine, Luzhou, China; ^3^ Department of Oncology, Affiliated Hospital of Southwest Medical University, Luzhou, China; ^4^ Department of Oncology and Hematology, Hospital (T.C.M) Affiliated to Southwest Medical University, Luzhou, China

**Keywords:** circRNA, oxidative stress, ROS, free radicals, biomarker

## Abstract

Oxidative stress caused by an imbalance between the production and elimination of reactive metabolites and free radicals can lead to the development of a variety of diseases. Over the past years, with the development of science and technology, circular RNA (circRNA) has been found to be closely associated with oxidative stress, which plays an important role in the process of oxidative stress. Currently, the understanding of circRNAs in the mechanism of oxidative stress is limited. In this review, we described the relationship between oxidative stress and circRNAs, the circRNAs related to oxidative stress, and the role of circRNAs in promoting or inhibiting the occurrence and development of diseases associated with the oxidative stress system.

## Introduction

**FIGURE 1:**
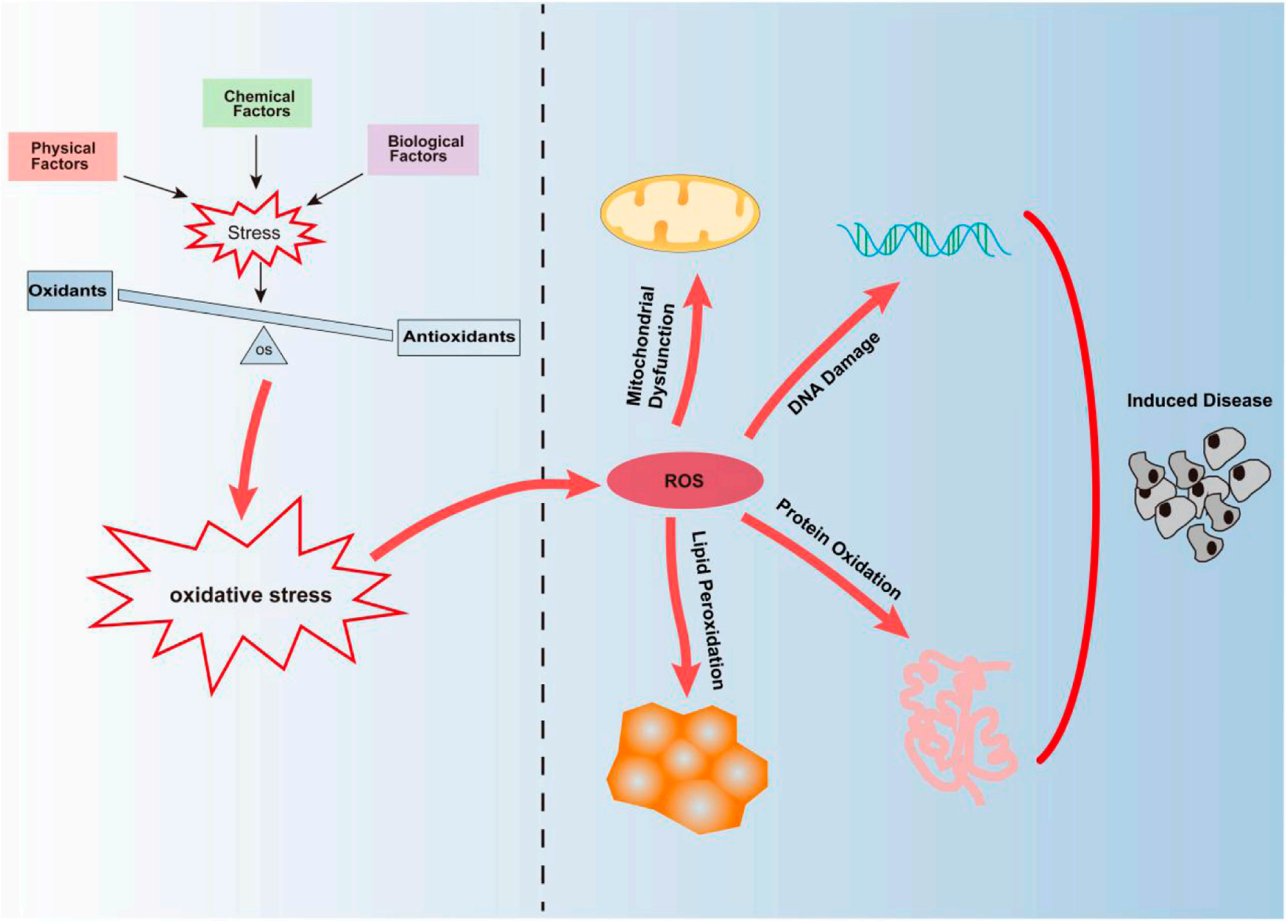
Oxidative stress is mainly caused by the imbalance between oxidation and antioxidant defense mechanisms caused by physical, chemical, and biological factors. Continuous oxidative stress can lead to the accumulation of ROS leading to mitochondrial dysfunction, DNA damage, protein oxidation, and lipid peroxidation, thus inducing the occurrence of diseases.

Oxidative stress is caused by an imbalance between the production of reactive oxygen species (ROS) and the antioxidant capacity (Birben et al., 2012). It is a negative effect produced by free radicals in the body. Persistent oxidative stress leads to the accumulation of ROS, destroying macromolecular substances, leading to DNA damage and further mutation, and induction of cancer and other diseases (Schieber and Chandel, 2014; Pisoschi and Pop, 2015; Baek and Lee, 2016) (Figure 1). Studies have found that oxidative stress is caused by a variety of factors and is regulated in many ways. As the main regulator" of antioxidant reactions, Nrf2 regulates the expression of genes involved in oxidative stress. Nrf2 protects cells from oxidative damage by inducing the expression of antioxidant and detoxification enzymes, thus activating the oxidative stress defense system *in vivo* (Hybertson et al., 2011; Ryoo and Kwak, 2018). However, Nrf2 cannot effectively scavenge the free radicals (Wang X. et al., 2019). This is due to oxidative stress drives the cellular physiological regulation response to be highly complicated, it is mainly achieved through signal transduction, transcription factors (TFs), and non-coding RNAs (ncRNAs) (Lin, 2019).

With the advent of advanced sequencing technologies, thousands of circRNAs have been identified in eukaryotes. Most circRNAs are predominantly derived from protein-coding genes and consist of one or more exons (Kristensen et al., 2019). These RNAs exhibit a covalently closed loop structure and do not contain the 5′ cap and 3′ poly (A) tail (Yu et al., 2019). Many studies have focused on the use of circRNAs as potential biomarkers for disease. Increasing number of studies are reporting that circRNAs are closely related to oxidative stress (Zhang J. et al., 2019; Chen et al., 2019; Yu et al., 2019; Hanan et al., 2020b; Saaoud et al., 2021), indicating that circRNA may play a central role in the generation of oxidative stress, and that there is an urgent need to obtain a detailed understanding of this relationship.

Here, we mainly used the electronic database (PubMed) to conduct a literature review on the role of circRNAs with respect to the promotion or inhibition of the occurrence and development of oxidative-stress‒related diseases. To this end, we used the following search terms in English: circRNA, circular RNA, non-coding RNA, oxidative stress, reactive oxygen species, ROS, free radicals, hypoxia, temperature, ionizing radiation, ultraviolet radiation, heavy metals, environmental toxins, medicine, nutrition, inflammation, and endoplasmic reticulum stress. There were no restrictions on the publication date. We reviewed the role of circRNAs in the generation of oxidative stress. Most importantly, we reviewed the current research progress on circRNA and oxidative-stress‒related diseases and described the potential mechanisms underlying the generation of oxidative stress to enable the discovery of potential biomarkers related to the disease.

## Factors Affecting Oxidative Stress

### Physical Factors

Physical factors that induce oxidative stress include hypoxia, temperature, ionizing radiations, and ultraviolet rays. An imbalance between oxygen supply and demand can result in the induction of hypoxia (Semenza, 2001). Oxygen is the ultimate acceptor of high-energy electrons produced during different metabolic processes. Under physiological conditions, a small part of the oxygen that is not reduced during aerobic metabolism gives rise to ROS (Torres-Cuevas et al., 2017). Hypoxic conditions are conducive to the increase in ROS levels and generation of oxidative stress and induce the accumulation of ROS, a phenomenon that leads to oxidative damage (Quinonez-Flores et al., 2016). Oxidative stress that occurs during intermittent or continuous cellular hypoxia may be related to the development of hypertension, cardiovascular and metabolic disorders, and respiratory diseases. The imbalance between ROS generation and clearance is the core pathological mechanism associated with hypoxia (Pialoux and Mounier, 2012). Temperature is one of the causative factors of oxidative stress; oxidative damage can be induced when the body exposed to either continuous high temperatures or low temperatures (Venditti et al., 2010; Blagojevic et al., 2011; Rhoads et al., 2013; Klarod et al., 2015; Xue et al., 2018). Cells exposed to ionizing radiation are prone to oxidative stress. Chemical and biological changes occur in cells upon irradiation with ionizing radiation or in response to the radiolysis of cellular water (Azzam et al., 2012). These changes are not just restricted to the cells exposed to the ionizing radiations but instead also affect their offspring (Mothersill and Seymour, 2004; Spitz et al., 2004; Prise and O'Sullivan, 2009). The progeny of the bystander cells also exhibit perturbations in oxidative metabolism and extensive oxidative damage, including phenomena, such as protein carbonylation, lipid peroxidation, spontaneous gene mutations, and increased tumor transformation (Buonanno et al., 2011). Appropriate irradiation with ultraviolet rays can result in the induction of vitamin D synthesis, but long-term exposure to ultraviolet light can result in the development of skin cancer, malignant melanoma, and other diseases. ROS produced by ultraviolet radiation represents one of the mechanisms through which ultraviolet light exerts its harmful effects on human health (De Jager et al., 2017). The skin is highly susceptible to continuous ultraviolet radiation, which stimulates melanin synthesis and leads to pigmentation, epidermal melanocytes are susceptible to high ROS levels, induced in response to excessive sunlight. If the increase in ROS levels is sufficient to disrupt homeostasis, malignant transformation might occur (Picardo et al., 1996; Denat et al., 2014).

### Chemical Factors

Chemicals present in the environment, drugs, food, and other substances can cause oxidative stress in the human body through certain mechanisms, which in turn trigger the development of oxidative-stress‒related diseases. Oxidative stress is known to be responsible for more than 200 diseases, including bacterial, viral, and parasitic infections, autoimmune diseases, malignant tumors, atherosclerosis, diabetes, kidney diseases, skin diseases, and neurodegeneration (Pisoschi and Pop, 2015; Baek and Lee, 2016). Pesticides, dioxins, paraquat, quinones, heavy metals, PM2.5, and other toxic compounds in the environment can cause problems with the physiological response of the body and further damage the biological macromolecules, leading to the excessive accumulation of ROS and subsequent oxidative stress (Ahmad, 1995; Yoshida and Ogawa, 2000; Valko et al., 2005; Kukongviriyapan et al., 2016; Guo et al., 2017; Winiarska-Mieczan, 2018). Drugs induce an increase in ROS levels, which in turn triggers the generation of oxidative stress. For example, glucocorticoids can indirectly induce oxidative stress by consuming antioxidant molecules or inhibiting the activity of antioxidant enzymes (Bjelakovic et al., 2007; Almeida et al., 2011; Feng and Tang, 2014). Cyclosporin A induces mitochondrial Ca^2+^ levels, increases oxidative stress and ROS production, and inhibits mitochondrial glucose metabolism and ATP production (Serkova et al., 2004). Insufficient or excessive nutrient intake can also lead to the generation of oxidative stress, and low intake or impaired availability of dietary antioxidants can weaken the antioxidant system (Sies et al., 2005; Saha et al., 2017).

### Biological Factors

Biological factors contribute substantially to oxidative stress. The accumulation of ROS, inflammation, and endoplasmic reticulum (ER) stress is the main biological factors that contribute to oxidative stress. Mitochondria are the main sites of oxygen metabolism, and ROS are the by-products of oxygen consumption and cell metabolism (Zorov et al., 2014). ROS are oxygen-containing derivatives comprising extremely unstable free radicals (Dores et al., 1990). Under normal physiological conditions, the intracellular level of ROS is stably maintained to prevent cell damage. Non-enzymatic molecules or antioxidant enzymes can specifically promote the elimination of ROS (Liou and Storz, 2010). The increase in ROS content leads to the destruction of DNA, proteins, and lipids, resulting in the generation of oxidative stress.

During inflammation, mast cells and leukocytes are recruited to the injury site, resulting in the induction of a “respiratory burst”. Increased oxygen uptake at the injury site leads to increased production and accumulation of ROS (Coussens and Werb, 2002). Inflammation and oxidative stress are closely linked (Rivera et al., 2017), and inflammation can result in the generation of a hypoxic microenvironment and induction of mitochondrial dysfunction, thereby triggering oxidative damage. Hypoxia promotes inflammation by activating HIF-1 and NF-κB, and inflammatory cells release a large amount of ROS, thereby causing excessive oxidative damage (Mcgarry et al., 2018). Studies have shown that changes in the redox homeostasis of the ER are sufficient to induce ER stress, which in turn can stimulate the production of ROS in the ER and mitochondria (Cao and Kaufman, 2014). Reports have revealed a correlation between ER stress and oxidative stress (Zhang and Kaufman, 2008).

## The Effect of Reactive Oxygen Species on Cell Physiology

ROS are produced by oxidases and eliminated by a scavenging system, which comprises enzymatic or non-enzymatic reactions. An imbalance between the generation and removal of ROS results in increased ROS levels (Sun et al., 2020). Continuously elevated levels of ROS promote the development of cardiovascular diseases, various cancers, and neurodegenerative diseases (Yan et al., 2013; Sabharwal and Schumacker, 2014; Panth et al., 2016; Moloney and Cotter, 2018; Senoner and Dichtl, 2019). Under physiological conditions, the balance between ROS generation and clearance is stringently controlled (Zorov et al., 2014). Excessive ROS levels can result in reduced energy metabolism, deregulation of signal transduction and cell cycle, mutations in transport-related genes—and by extension, reduced biological activity—immune cell activation, and inflammation (Newsholme et al., 2016). ROS at appropriate concentrations plays an important regulatory and intermediary role in signal transduction, thereby protecting cells from oxidative stress and restoring “redox homeostasis.” In higher organisms, ROS also function as signaling molecules to enable physiological functions, such as regulation of vascular tension and production of erythropoietin; they serve as ligands for various membrane receptors in various physiological processes (Droge, 2002).

ROS are key regulators of intracellular signaling pathways (Finkel, 2011). The process of ROS production by the NADPH oxidase 2 (NOX2) complex of phagocytes is referred to as “respiratory burst”. It has always been thought that ROS are pro-inflammatory in nature. However, increasing numbers of studies indicate that ROS produced by the NOX2 complexes exert anti-inflammatory effects. Therefore, the production of ROS by phagocytes is considered an important part of innate immunity (Hultqvist et al., 2009; Sareila et al., 2011; Li et al., 2016). The ER and mitochondria are the main sources of ROS, and play a crucial role in the regulation of apoptosis and autophagy (Kaminskyy and Zhivotovsky, 2014). The use of antioxidants to interfere with the physiological functions of ROS may result in adverse effects. Nrf2 is the main transcription factor that regulates oxidative stress. It protects cells from oxidative damage by inducing the expression of antioxidant and detoxifying enzymes, thereby activating the antioxidant defense system of the body (Ryoo and Kwak, 2018). However, overexpression of Nrf2 results in increased expression of several molecules involved in intracellular redox balance maintenance, phase II detoxification, and cellular translocation, all of which can provide cancer cells with a growth advantage and induce the development of resistance against chemotherapy (Lau et al., 2008). Nrf2 activation in cancer cells not only leads to the development of drug resistance but also promotes their proliferation (Ohta et al., 2008). We can leverage the physiological properties of ROS to treat diseases. For example, targeting cancer cells through ROS-mediated mechanisms represents a new direction for cancer treatment in the future (Trachootham et al., 2009).

## CircRNA Regulates Reactive Oxygen Species Production

ROS function as signaling molecules that cause physiological responses (Dupre-Crochet et al., 2013). Excessive ROS can induce oxidative stress, damage macromolecules, cause inflammation, and induce the accumulation of genetic mutations. Previous studies have reported a relationship between non-coding RNAs and oxidative stress. MicroRNAs might serve as highly effective biomarkers and therapeutic targets for oxidative-stress‒related diseases (Banerjee et al., 2017). Oxidative-stress‒related long non-coding (lncRNAs)—as potential biomarkers and drug targets—may enable the development of new strategies for disease diagnosis and treatment (Wang X. et al., 2019). Compared with microRNAs and lncRNAs, circRNAs are more stable and resistant to exonuclease degradation. Further, as they function as sponges to regulate the effects of microRNAs (Saaoud et al., 2021), circRNAs can also play an important role in mediating the generation of oxidative stress. The expression of circRNAs is regulated by oxidative stress. They mediate the production of ROS and promote ROS-induced cell death, apoptosis, and inflammation (Saaoud et al., 2021). Studies have shown that L02 cells exhibit substantially increased circRNA-4099 levels in response to hydrogen peroxide (H_2_O_2_)-induced oxidative stress, and the generated circRNA-4099 further triggers the activation of the keap1/Nrf2-p38 MAPK cascade by enhancing the phosphorylation of related proteins, downregulating miR-706 expression, and enhancing the intracellular H_2_O_2_ levels, thereby promoting apoptosis, ROS generation, and fibrosis (Li et al., 2020b). The circPRKCI-miR-545/589-E2F7 axis plays an important role in mediating H_2_O_2_-induced neuronal damage. H_2_O_2_ downregulates the expression of circPRKCI in neuronal cells, leading to miR-545/589 accumulation and downregulation of E2F7 expression. Heterotopic overexpression of circPRKCI, or transfection with miR-545/589 inhibitor, can effectively mitigate H_2_O_2_-induced neuronal death and apoptosis (Cheng et al., 2019).

## Research Progress on CircRNAs and Oxidative-Stress‒Related Diseases

The development of high-throughput sequencing technology has enabled scientists to discover thousands of circRNAs, which play a key role in biological processes (Hansen et al., 2013; Thomas and Saetrom, 2014). In addition to their miRNA-sponging functions, circRNAs are involved in phenomena, such as transcription, splicing, and translation, additionally, they also serve as protein decoys. (Legnini et al., 2017; Panda et al., 2017). CircRNAs can be divided into the following categories: exon circRNAs (ecircRNAs), circular intron RNA (ciRNA), exon-intron circRNA (EIciRNA), and tRNA intron circRNAs (tricRNAs) based on sequence composition. Further, circRNAs can also be categorized as cytoplasmic and nuclear circRNAs based on their localization in the cell (Chen et al., 2015; Li et al., 2015; Zhao et al., 2019). As circRNAs can be categorized into different subtypes, their possible mechanisms of action are highly complicated. For example, the function as a transcriptional regulator may be unique to EIciRNA, but might not be necessarily extrapolated to other circRNAs (Chen et al., 2015), this makes circRNAs promising disease biomarkers.

Recently, it has been reported that circRNAs play a role in promoting or inhibiting the occurrence and development of diseases related to oxidative stress, such as cardiovascular diseases and Parkinson’s disease (Li et al., 2018; Hanan et al., 2020a). Under physiological conditions, ROS affect the vascular function by regulating various redox-sensitive signaling pathways (Saaoud et al., 2021). Oxidative stress triggers the development of cardiovascular diseases by inducing vascular endothelial dysfunction and inflammation (Steven et al., 2019). Some studies have shown that the expression of some circRNAs is regulated by oxidative stress, and that these circRNAs mediate the production of ROS, and promote ROS-induced cell death and inflammation (Saaoud et al., 2021). Previous studies have reported that the expression of cZNF609 is significantly upregulated *in vivo* and *in vitro* in response to high glucose and hypoxia stress. Silencing of cZNF609 results in reduced loss of retinal blood vessels and inhibition of pathological angiogenesis *in vivo*. Further, cZNF609 silencing results in increased endothelial cell migration and tube formation and protection of endothelial cells from oxidative and hypoxic stresses *in vitro* (Liu et al., 2017). CircHIPK3 is known to regulate lipopolysaccharide (LPS)-induced oxidative stress and inflammation *in vivo* and *in vitro*, upregulation of this circRNA is known to protect against LPS-induced oxidative damage and myocardial inflammation, and inhibit apoptosis (Fan et al., 2020). CircHIPK3 is a circular RNA whose expression is known to be significantly upregulated in hypoxic exosomes. This RNA is known to reduce oxidative stress-induced dysfunction of cardiac microvascular endothelial cells (CMVECs) via the miR-29a/IGF-1 axis (Wang Y. et al., 2019).

**FIGURE 2:**
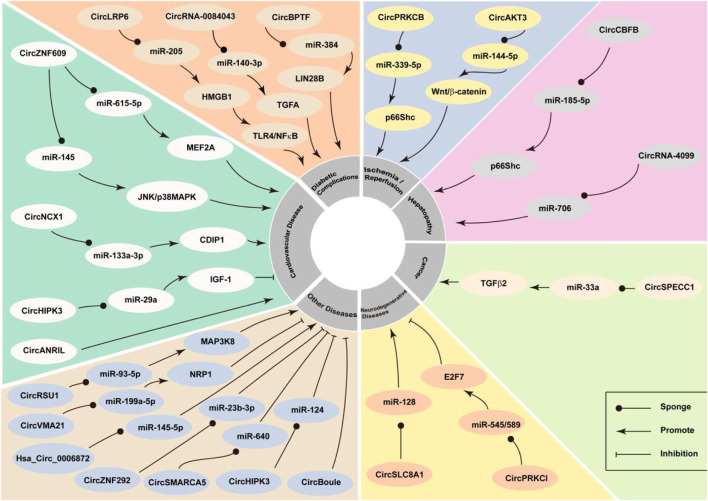
CircRNA participates in oxidative stress and has the effect of increased oxidative stress or anti-oxidation, leading to the development or inhibition of oxidative stress-related diseases.

However, CircNFIX is another circular RNA whose expression is known to be significantly downregulated in cardiomyocytes in response to oxidative stress; this RNA could serve as a proapoptotic factor during cardiomyocyte apoptosis. Thus, circNFIX has the potential to serve as a biomarker and therapeutic target for myocardial infarction (Cui et al., 2020). CircHIPK3 is known to be significantly downregulated in response to H_2_O_2_-induced oxidative stress in OB-6 cells and primary human osteoblasts. Overexpression of circHIPK3 alleviates the H_2_O_2_-induced cell death (Liang J. et al., 2020). High glucose (HG) induces the downregulation of circACR in RSC96 cells. CircACR may downregulate the expression of miR-145-3p and promote the activation of the PI3K/AKT/mTOR pathway to alleviate HG-induced apoptosis, autophagy, and ROS generation in RSC96 cells (Liu et al., 2019). Heat stress may enhance the generation of oxidative stress (Slimen et al., 2014). Studies have revealed that circBoule regulates the expression of heat shock proteins under conditions of heat stress to protect male fertility in animals (Figure 2) (Gao et al., 2020). These studies indicate that circRNAs play an important role in the development of oxidative-stress‒related diseases and that they can be used as a potential target for disease diagnosis and treatment.

## CircRNAs Mediate the Generation of Oxidative Stress

Studies have reported that circRNAs play a role in the development of oxidative-stress‒related diseases, and that is possibly mediated via the interaction between circRNAs and the key genes of the oxidative stress system (Figure 2). CircRNAs related to oxidative stress are summarised in Table 1.

**TABLE 1:** CircRNA regulates oxidative stress.

CircRNA	Target	Function	References
CircZNF609	miR-615-5p; miR-145	Promote oxidative stress	Liu et al. (2017), Ge and Gao (2020)
CircLRP6	miR-205	Promote oxidative stress	Chen et al. (2019)
CircNCX1	miR-133a-3p	Promote oxidative stress	Ruan et al. (2020)
CircSLC8A1	miR-128	Oxidative stress-induced Parkinson’s disease is a major participant	Hanan et al. (2020b)
CircHIPK3	miR-29a; miR-124	Antioxidant	Wang et al. (2019b), Liang et al. (2020a)
CircPRKCB	miR-339-5p	Induced ROS accumulation	Feng et al. (2020)
CircSMARCA5	miR-640	Inhibit oxidative stress	Cai et al. (2020)
CircRNA_0084043	miR-140-3p	Promote oxidative stress	Li et al. (2020a)
CircANRIL		Promote oxidative stress	Shi et al. (2020a)
CircBPTF	miR-384	Promote oxidative stress	Zhang and Sui (2020)
CircAKT3	miR-144-5p	Promote oxidative stress	Xu et al. (2020)
CircSPECC1	miR-33a	Promote oxidative stress	Zhang et al. (2020a)
Hsa_circ_0006872	miR-145-5p	Promote oxidative stress	Xue et al. (2021)
CircVMA21	miR-199a-5p; miR-9-3p	Inhibit oxidative stress	Shi et al. (2020b), Li et al. (2021)
CircPRKCI	miR-545; miR-589	Inhibit oxidative stress	Cheng et al. (2019)
CircRSU1	miR-93-5p	Promote oxidative stress	Yang et al. (2021)
CircCBFB	miR-185-5p	A key mediator of oxidative stress	Wang et al. (2020)
CircRNA-4099	miR-706	Promote oxidative stress	Li et al. (2020b)
CircZNF292	miR-23b-3p	Regulate antioxidant genes	Liang et al. (2020b)

### CircZNF609

CircZNF609 originates as the result of the circularization of the second exon of the host gene. A 753-nucleotide open reading frame exists in circZNF609, i.e., from the AUG of the host gene to the stop codon generated by 3 nt after splicing (Legnini et al., 2017). It has been reported that circZNF609 is an important intermediary for the generation of oxidative stress (Ge and Gao, 2020). CircZNF609 silencing may protect HaCaT cells from H_2_O_2_-induced oxidative stress via the regulation of the miR-145-triggered JNK and p38MAPK signaling pathways (Ge and Gao, 2020). There is evidence that circZNF609 acts as an endogenous sponge for miR-615-5p thereby resulting in MEF2A expression. Overexpression of MEF2A can rescue the effects of circZNF609 silencing on endothelial cell migration, tube formation, and apoptosis. Silencing of circZNF609 can result in the protection of endothelial cells from oxidative and hypoxic stresses and can be used as a potential strategy for the treatment of oxidative-stress‒related vascular diseases (Liu et al., 2017).

### CircLRP6

CircLRP6 is upregulated in mesangial cells treated with HG and acts as a molecular sponge for miR-205 to regulate HG-induced mesangial cell damage. In addition, overexpression of miR-205 or knockdown of circLRP6 results in the inhibition of the NF-κB pathway. As an intermediary of the newly discovered circLRP6-miR-205-HMGB1-TLR4/NF-κB regulatory network, circLRP6 regulates HG-induced mesangial cell proliferation, oxidative stress, extracellular matrix accumulation, and inflammation by the following mechanisms: sponging miR-205, upregulating HMGB1, and activating the TLR4/NF-κB pathway (Chen et al., 2019). circLRP6 can serve as an important therapeutic biomarker for diabetic nephropathy.

### CircNCX1

CircNCX1 is transcribed from the gene coding for sodium/calcium exchange protein 1 (*NCX1*). This RNA acts as a sponge for miR-133a-3p, thereby inhibiting miR-133a-3p-mediated regulation of CDIP1 during myocardial ischemia-reperfusion (MI/R) injury and promoting cardiomyocyte apoptosis (Ruan et al., 2020). circNCX1 is highly expressed in cardiomyocytes. A study revealed a new regulatory pathway, involving circNCX1, miR-133a-3p, and CDIP1, all of which are related to cardiomyocyte apoptosis. This pathway can be used as a potential therapeutic target for ischemic heart disease (Li et al., 2018).

### CircSLC8A1

CircSLC8A1 comprises an 1,832-basepair exon of the gene encoding solute carrier family 8 member 1 (*SLC8A1*), which is predominantly expressed in the cytoplasm of eukaryotic cells. As this RNA harbors multiple miRNA-binding sites (Dezfulian et al., 1987), it is related to cancer (Lu et al., 2019; Zhu et al., 2021), cardiovascular disease (Lim et al., 2019; Tian et al., 2021), and Parkinson’s disease (Hanan et al., 2020b). Some studies have also revealed that circSLC8A1 plays a role in the generation of oxidative stress (Hanan et al., 2020b). SH-SY cells (human neuronal cell line) exposed to paraquat-induced oxidative stress exhibited increased expression of circSLC8A1. However, cells treated with the neuroprotective antioxidant modifier, simvastatin, were characterized by decreased expression of circSLC8A1. Oxidation *per se* may result in the increased cyclization of circSLC8A1 or decreased degradation of circRNA in neurons. Therefore, we can speculate that circSLC8A1 is an important molecule in Parkinson’s disease (Hanan et al., 2020b).

### CircHIPK3

CircHIPK3 is a highly expressed stable RNA that is derived from exon 2 of *HIPK3*. This RNA was reported to bind to nine miRNAs that among themselves harbored 18 potential binding sites (Zheng et al., 2016). CircHIPK3 is known to be involved in the development and progression of cancer (Zeng et al., 2018; Zhang Y. et al., 2019). It can serve as a novel therapeutic target and diagnostic and prognostic biomarker in different cancer types (Zhang Y. et al., 2020). Recently, there has been a new understanding regarding the role of circHIPK3 as a potential target of oxidative stress. It has been demonstrated that overexpression of circHIPK3 significantly reduced apoptosis and the oxidative status in CMVECs exposed to oxidative stress. CircHIPK3 modulates oxidative damage in CMVEC via the miR-29a/IGF-1 axis. Studies demonstrated that circHIPK3 has an antioxidant function and is a potential target for protecting CMVECs from oxidative stress (Wang Y. et al., 2019). Several studies have investigated the expression and potential function of circHIPK3 in the background of H_2_O_2_-induced oxidative damage in human osteoblasts. Downregulation of miR-124 expression by circHIPK3 may mediate H_2_O_2_-induced toxicity in human osteoblasts. Targeting the circHIPK3-miR-124 cascade serves as a strategy to protect human osteoblasts from oxidative damage (Liang J. et al., 2020).

### CircPRKCB

p66Shc plays a key role in ROS production (Napoli et al., 2003). CircPRKCB promotes the accumulation of ROS in response to intestinal I/R injury by sponging miR-339-5p and upregulating the expression of p66Shc. The key role of the circPRKCB/miR-339-5p/p66Shc signaling pathway in regulating I/R intestinal oxidative stress has been demonstrated. This pathway may serve as a potential therapeutic target for intestinal I/R injury (Feng et al., 2020).

### CircSMARCA5

CircSMARCA5 (also known as hsa_circ_0001445) originates from exons 15 and 16 of *SMARCA5*, which encodes a specific chromatin remodeling protein SNF2H that regulates chromatin structure to promote DNA transcription and repair (Cai et al., 2019). Overexpression of hsa_circ_0001445 can inhibit the inflammatory response of oxidized low-density lipoprotein-treated human umbilical vein endothelial cells and reduces the expression of inflammatory factors and the level of oxidative stress (Cai et al., 2020).

### CircRNA_0084043

The deletion of circRNA_0084043 protects against HG-induced ARPE-19 cell damage by sponging miR-140-3p to induce the expression of transforming growth factor-alpha. The deletion of circRNA_0084043 significantly improved the cell survival rate and inhibited HG-induced apoptosis. In addition, circRNA_0084043 knockout significantly reduced oxidative stress, decreased the malondialdehyde content, and increased superoxide dismutase and glutathione peroxidase activities. Silencing of circRNA_0084043 effectively inhibited the HG-induced inflammatory response; this might serve as a potential therapeutic strategy for diabetic retinopathy (Li et al., 2020a).

### CircANRIL

CircANRIL is a circular antisense non-coding RNA transcribed from the INK4 locus (atherosclerotic cardiovascular disease locus) on chromosome 9p21 (Holdt et al., 2016). High expression of circANRIL in the human vascular tissue is known to be related to reduced severity of coronary artery disease (CAD). Inhibition of circANRIL expression in CAD can reduce vascular endothelial damage, oxidative stress, and inflammation, and can therefore serve as a new diagnostic and treatment strategy (Shi P. et al., 2020).

### CircBPTF

CircBPTF (hsa_circ_0000799) is a novel circRNA that is spliced by reverse splicing from exons 21 to 27 of the gene encoding bromodomain PHD finger transcription factor (*BPTF*) (Bi et al., 2018). Knockout of circBPTF can improve the activity of human umbilical vein endothelial cells (HUVECs) and inhibit HG-induced apoptosis, inflammatory response, and oxidative stress. In conclusion, CircBPTF knockout acts via the miR-384/Lin28b axis in HUVECs, which has a protective effect on HG-induced inflammatory damage and oxidative stress, thereby providing guidance for future treatment strategies for diabetes-related vascular complications (Zhang and Sui, 2020).

### CircAKT3

CircRNA AKT3 (circAKT3) is a novel RNA derived from *AKT3* (Zang et al., 2021), It mediates renal I/R (RI/R) damage by regulating oxidative stress and the miR-144-5p/Wnt/β-catenin pathway. CircAKT3 can promote apoptosis and activate the Wnt/β-catenin signaling pathway, thereby serving as a new target for the treatment of RI/R injury (Xu et al., 2020).

### CircSPECC1

CircSPECC1 (hsa_circ_0000745) is a novel circRNA, which has been reported to be significantly downregulated in H_2_O_2_-treated HCC cells. Knocking out circSPECC1 in the background under H_2_O_2_ treatment, inhibited the proliferation of HCC cells and promoted their apoptosis. In addition, circSPECC1 inhibits miR-33a expression through direct interaction, and miR-33a inhibitors can partially reverse the effects of circSPECC1 knockdown on proliferation and apoptosis. CircSPECC1 acts as a miR-33a sponge, regulating TGFβ-2 and autophagy under conditions of oxidative stress to promote the occurrence of HCC tumors. These findings may provide potential treatment strategies for patients with liver cancer (Zhang B. et al., 2020).

### Hsa_circ_0006872

Hsa_circ_0006872 levels were increased in patients with chronic obstructive pulmonary disease and negatively correlated with miR-145-5p levels. Hsa_circ_0006872 promotes apoptosis, inflammation, and oxidative stress triggered by cigarette smoke extract in HPMECs and BEAS-2B cells by regulating the miR-145-5p/NF-κB pathway. Hsa_circ_0006872 silencing inhibits CSE-induced cell damage by regulating miR-145-5p (Xue et al., 2021).

### CircVMA21

CircVMA21 (also known as hsa_circ_0091702) is derived from the gene encoding vacuolar ATPase assembly factor (*VMA21*) (Cheng et al., 2018). Studies have found that circVMA21 can decrease apoptosis in rats to reduce intervertebral disc degeneration and protect WI-38 cells from LPS-induced damage. Overexpression of circVMA21 inhibited LPS-induced, apoptosis, inflammation, and oxidative stress in THP-1 cells. The results showed that circVMA21 regulates the expression of NRP1 by sponging miR-199a-5p (Li et al., 2021). CircVMA21 plays an important role in affecting acute renal lesions related to sepsis by regulating the miR-9-3p/SMG1/inflammatory axis and oxidative stress (Shi Y. et al., 2020).

### CircPRKCI

CircPRKCI, which is a 1,484 bp long circRNA, is spliced in the opposite direction from the two exons (15 and 16) of *PRKCI* located at 3q26.2 and plays a key role in the occurrence and development of a variety of digestive system tumors (Zhang X. et al., 2019; Qi et al., 2019). In cultured SH-SY5Y nerve cells, H_2_O_2_ downregulated the expression of circPRKCI, leading to the accumulation of miR-545 and miR-589, but the expression of its target transcription factor E2F7 decreased. Importantly, ectopic overexpression of circPRKCI attenuated H_2_O_2_-induced cytotoxicity. The results of this study showed that the imbalance in the PRKCI-miR-545/589-E2F7 axis mediates H_2_O_2_-induced neuronal damage. Targeting this new cascade may be a new strategy for protecting neurons against oxidative damage (Cheng et al., 2019).

### CircRSU1

CircRSU1 was significantly upregulated in human articular chondrocytes and osteoarthritis (OA) articular cartilage treated with H_2_O_2_. CircRSU1 expression is induced by IL-1β and H_2_O_2_ stimulation and subsequently occurs through the MEK/ERK1/2 and NF-κB pathways. Studies have shown that the circRSU1-miR-93-5p-MAP3K8 axis plays a critical role in the regulation of circRSU1 in oxidative stress and ECM homeostasis in human chondrocytes. This axis affects OA through oxidative stress regulation and can be used as a potential target for OA treatment (Yang et al., 2021).

### CircCBFB

CircCBFB is a circRNA derived from the core-binding factor-beta (*CBFB*) gene locus and is highly expressed in acetaminophen (APAP) treatment. p66Shc is the main regulator of mitochondrial ROS and a key mediator of oxidative stress in liver cells. CircCBFB acts as a miR-185-5p sponge and regulates APAP-induced liver damage by targeting p66Shc. This provides a potential therapeutic target for APAP-induced liver damage (Wang et al., 2020).

### CircRNA-4099

CircRNA-4099 (hsa_circRNA_100759) is located in exon 11 (Wang et al., 2018). Hepatic fibers are always induced by oxidative stress. H_2_O_2_ induces the L02 cell line to inhibit viability and promote cell apoptosis, ROS production, and cell fibrosis, as well as the keap1/Nrf2 and p38MAPK cascades. H_2_O_2_ stimulated circRNA-4099, and an increased level of circRNA-4099 enhanced H_2_O_2_-induced damage by inhibiting miR-706. CircRNA-4099 inhibits miR-706 expression by triggering the keap1/Nrf2 and p38MAPK pathways in L02 cells, aggravating H_2_O_2_-induced damage, and provides a promising therapeutic target for hepatitis or hepatic fibrosis (Li et al., 2020b).

### CircZNF292

Oxidative stress is one of the key factors in the pathogenesis of age-related cataracts (ARC) (Berthoud and Beyer, 2009). The level of circZNF292 decreased significantly and that of miR-23b-3p increased significantly in ARC. CircZNF292 acts as a competitive endogenous RNA and regulates the expression of antioxidant genes by competing with miR-23b-3p. The results of this study showed that circZNF292, a circRNA which is downregulated in the anterior lens capsule of patients with ARC, can act as a miR-23b-3p sponge and participate in the resistance to oxidative damage and anti-apoptosis of lens epithelial cells, thereby serving as a potential biomarker for the prevention and treatment of ARC (Liang S. et al., 2020).

## Conclusion

In recent years, scientists have become increasingly enthusiastic about circRNA research. Nonetheless, the mechanisms of action of circRNAs have still not been completely elucidated. In this review, we focused on circRNAs related to oxidative stress, introduced their mechanisms of action, and provided potential therapeutic targets for oxidative-stress‒related diseases, but problems still exist. First, with respect to the mechanism of action of circRNA in oxidative-stress‒related diseases, most studies have focused on the circRNA-miRNA-mRNA regulatory networks, but there is very little research on the interaction between circRNAs. Future research needs to systematically study the mechanism of action of circRNA in the context of oxidative-stress‒related diseases to clarify the relationship between circRNA and oxidative-stress‒related diseases. In addition, because circRNA is categorized into multiple subtypes, different functional mechanisms are possible. In future studies, the roles of different subtypes of circRNAs and oxidative-stress‒related diseases should be emphasized. Finally, as promising biomarkers and drug targets, circRNAs may provide a new direction for the diagnosis and treatment of diseases. Future research should focus on the precise mechanism of action of circRNA with respect to the regulation of oxidative stress.
